# Amaranth selective hydrolyzed protein influence on sourdough fermentation and wheat bread quality

**DOI:** 10.1002/fsn3.2618

**Published:** 2021-10-13

**Authors:** Nayereh Karimi, Fariba Zeynali, Mahmoud Rezazad Bari, Mehdi Nikoo, Forogh Mohtarami, Mahdi Kadivar

**Affiliations:** ^1^ Department of Food Science and Technology Faculty of Agriculture and Natural Resources Urmia University Urmia Iran; ^2^ Department of Pathobiology and Quality Control Artemia and Aquaculture Research Institute Urmia University Urmia Iran; ^3^ Department of Food Science and Technology College of Agriculture Isfahan University of Technology Isfahan Iran

**Keywords:** amaranth, bread properties, fermentation, protein hydrolysates, sourdough

## Abstract

Amaranth selective hydrolyzed protein (ASPH) may improve sourdough properties and bread quality. In this regard, this study focused on investigating the influence of protein hydrolysates on sourdough fermentation and bread properties. Based on the findings, ASPH further increased *Lactobacillus plantarum* and *Saccharomyces cerevisiae* growth in sourdough compared with amaranth protein isolates and amaranth flour. ASPH at 5 g/kg resulted in sourdough with higher pH and total titratable acidity (TTA) after 20 h of fermentation at 30°C. The prepared sourdough using APH (S‐ASPH) at 3 g/kg increased the specific volume (4.57 ml/g) and TTA (4.76 ml) while decreasing water activity, hardness, cohesiveness, and chewiness of the bread (S‐ASPH‐B) compared with the control. Moreover, transition temperature and enthalpy reduced whereas sensory properties and shelf life represented an increase with S‐ASPH addition. Overall, the obtained data indicated the improvement of bread quality by S‐ASPH sourdough.

## Graphical abstract

Our results indicated the improvement of bread quality by sourdough prepared with amaranth protein hydrolysates. ASHP might be useful as natural ingredients of sourdough and wheat bread.

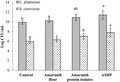

## 1 INTRODUCTION

Sourdough has been traditionally used as a natural starter for making fermented baked products as an alternative to bakery's yeast and chemical leavening (Arora et al., 2021). Due to unique microbial flora and functionality, sourdough influences dough and bread by increasing the loaf volume, slowing staling and improving flavor and hygienic properties compared to conventional breads (Olojede et al., 2020; Yu et al., 2019). In addition, the acidification of dough affects its microbial growth and enzymes, dough rheology, and bread sensory attributes, and shelf life (Jagelaviciute & Cizeikiene, 2021). Yeasts and lactic acid bacteria (LAB) are the main components of sourdough microbial composition and have an important role in acidification, aroma formation, and bread's unique characteristics (Fraberger et al., 2020; Xi et al., 2020). Depending on the type of flour, the mixtures of carboxylic acids with antimicrobial properties are produced in sourdough (Jagelaviciute & Cizeikiene, 2021). Alpha‐amylase activity from the LAB acidification process mainly influence bread texture by producing low molecular weight dextrins decreasing the amylopectin associated retrogradation and interfering between gluten‐starch interactions during bread storage, thus lowering the firming rate (Falade et al., 2014).

The potential nutritional and biological significance of food proteins has driven the interest in the production of “functional” foods that are nutritious and healthy (Nikoo et al., 2021; Sarteshnizi et al., 2021; Sun et al., 2020). Amaranth (*Amaranthus hypochondriacus*) is a pseudocereal, which is considered as an alternative crop due to its nutritional (high quality proteins and low saturated fatty acids) and technological properties for the baking industry (Janssen et al., 2017). Amaranth grain consists of three major proteins such as albumins (40%), globulins (20%), and glutelins (25%–30%) with 2%–3% prolamin (Tamsen et al., 2018). Amaranth flour (AF) also has been used in many food products including cakes, muffins, breads, pan cakes, noodles, dumplings, baby purees, cookies, and nuggets (Jiménez et al., 2020; Miranda‐Ramos et al., 2019; Tamsen et al., 2018). Amaranth seeds or parts of it possess several biological functions such as antioxidant, antidiabetic, anti‐inflammatory, and antihyperlipidemic effects (Miranda‐Ramos et al., 2019). Moreover, amaranth protein hydrolysates have been suggested as a promising source of bioactive peptides with potential health benefits (Delgado et al., 2011; Mudgil et al., 2019; Tironi & Añón, 2010).

In recent years, the demand for foods containing fermented products is increasing (Olojede et al., 2020). During sourdough fermentation, LAB and yeast proteolytic activities produce bioactive peptides (Luti et al., 2020; Zhao et al., 2013). In this respect, sourdough fermentation is one of the traditional techniques for improving the nutritional and functional health properties of many foods (Francesca et al., 2019; Gänzle et al., 2007; Suo et al., 2021). The use of protein hydrolysates in sourdough may improve processing conditions with regard to the acidification rate, improved fermentation process, and bread properties. Given the above‐mentioned explanations, the current study aimed to evaluate the effects of ASPH on sourdough fermentation and bread properties and shelf life.

## 2 MATERIALS AND METHODS

### 2.1 Preparation of amaranth protein isolates

AF was obtained by the grinding (FP970 series, Multipro Excel, Kenwood Company) of dried amaranth seeds and sieved (100 µm sieve size) to obtain a fine powder. It was defatted using hexane (1:10 g/ml) under continuous stirring for 24 h at 4ºC (Ayala‐Niño et al., 2019). The flour of amaranth was dried at room temperature (24 ± 1ºC) for 24 h and then stored at 4ºC until used. The moisture content in AF was 6.7% and 7.3% before drying and after defatted, respectively. The total proteins‐rich fraction was solubilized at a pH value of 11.0 using 1 mol/L NaOH and then precipitated at pH ~5 by 1 mol/L HCl. After centrifugation at 4000 × *g* for 10 min at 4°C, the precipitate was freeze‐dried and referred to as the amaranth protein isolates (API).

### 2.2 Preparation of ASHP

The API (10 mg/ml) was mixed with distilled water and preheated at 50ºC for 30 min. Further, the pH was adjusted to 8.0 by 1 mol/L NaOH and hydrolysis was performed using Alcalase (Sigma‐Aldrich Co) at an enzyme to substrate ratio of 1:20 (g:g) according to previous research (Nikoo et al., 2019). After the specified reaction time (3 h), the enzyme was deactivated by heating at ~95ºC for 10 min and then cooled using iced water for 10 min. The hydrolysate solution was centrifuged (4000 g, 4ºC, 10 min) to obtain a clear supernatant and then freeze‐dried as well.

### 2.3 Effect of ASHP on the growth of *L. plantarum* and *S. cerevisiae*

ASPH was tested for its effect on the growth of *L*. *plantarum* PTCC (1896) and *S. cerevisiae* PTCC (5052). Hydrolysates (2 mg/ml) were added into test tubes containing 10^4^ cfu/ml bacteria or yeast cells. Next, the *L*. *plantarum* count was conducted by the pour plate technique on MRS agar (Merck) and *S*. *cerevisiae* count was grown on PDA (Merk) at 30°C for 48 h (Zhao et al., 2020).

### 2.4 Preparation of sourdough

Sourdough was prepared according to the method described by Yu et al. (2019) using ASHP. Sourdough composing of 0–5 g/100 g ASHP was prepared, referred to as S‐ASHP, and stored at 30ºC for 20 h.

### 2.5 Preparation of bread using S‐ASHP sourdough (S‐ASHP‐B)

The bread was prepared by mixing 100 g flour with salt (1/100 g), bread improver (N300, Nanafza Improver Co., Tehran, Iran, 1/100 g), yeast (1.5/100 g) and 20/100 g S‐ASHP sourdough (Katina et al., 2005), and 56 ml water (Katina et al., 2006). Furthermore, the prepared bread using common sourdough (CSB), which was provided without amaranth hydrolysate was considered as the control bread. The dough was allowed to proof for 30 min at 30ºC after mixing its ingredients. The dough was kept for final proofing at 30ºC and relative humidity of 85% for 90 min. Finally, the dough was baked for 15 min at 220ºC (Oven 1600, Mashhad Baking Industries Co.) and cooled at room temperature (25 ± 1ºC) for 1 h before being packed into polyethylene bags (Zip. Kip Co., Karaj, Alborz, Iran) according to a previous study (Meignen et al., 2001).

### 2.6 Sourdough properties

The counts of *L. plantarum* and *S. cerevisiae*, as well as pH, and TTA were determined at time 0 and 20 h of sourdough fermentation. The pH was determined using a pH meter after mixing sourdough with water (1:10 g:g), and TTA (expressed as mL of NaOH) was measured by the method of Katina et al. (2006).

### 2.7 Moisture and *a_w_*

The moisture of the bread crumb was determined after heating 5 g of a slice from the bread center at 105ºC for 12 h (AOAC, 2005), followed by measuring the water activity (a_w_) of fresh bread crumb using a Novasina *a_w_* Sprint TH500 (Pfäffikon, Switzerland) after equilibration at 25°C.

### 2.8 Bread texture properties

The texture of the bread without crust the (2 × 2 × 2 cm) was measured by a Texture Analyzer TA‐XT2i (Stable Microsystems) equipped with an aluminum 25‐mm diameter cylindrical probe. The measurement parameters were set as the distance of 5 mm, test speed of 0.5 mm/s, trigger force of 5 g, post‐test speed 10.00 mm/s, pretest speed 1.00 mm/s, and trigger type auto. The data were measured as force (N) versus time (s) curves. The hardness (g), springiness (cm), cohesiveness (g), and chewiness were recorded (Yu et al., 2019) as well.

### 2.9 Bread specific volume

The loaf volume was measured using AACC 10‐05 (AACC, 2001) according to Yu et al. (2019) and calculated by dividing the loaf volume by loaf weight.

### 2.10 Thermal properties of bread

These properties were determined using a differential scanning calorimeter (DSC) instrument (DSC PT 10, Linseis). Bread crumb (10 mg) was equilibrated at 25°C for 3 min before being heated at 10ºC/min to 100ºC (Torrieri et al., 2014). The denaturation peak temperature and enthalpy were calculated with TA software.

### 2.11 Shelf life of bread

Bread samples were kept at room temperature and mold growth was determined for up to 7 days by visual observations (Gerez et al., 2009).

### 2.12 Sensory analysis

The participants consisted of 80 people (40 males and 40 females), ages 22 to 54. The participants scored the bread samples using a scale from 1 to 9 (1 for dislike immensely and 9 for like immensely) as described by Karimi et al. (2021).

### 2.13 Statistical analysis

ANOVA tests were applied to analyze *L. plantarum* and *S. cerevisiae* growth, S‐ASHP pH and TTA data significant differences between the mean values were compared using Duncan's multiple range tests at *p* ≤ .05, and data were expressed as the mean ± standard deviation. The differences between S‐ASHP‐B and control bread properties were determined using an independent‐samples *t* test.

## 3 RESULT AND DISCUSSION

### 3.1 Effect of ASHP on the growth of *L. plantarum* and *S. cerevisiae*

**FIGURE 1:**
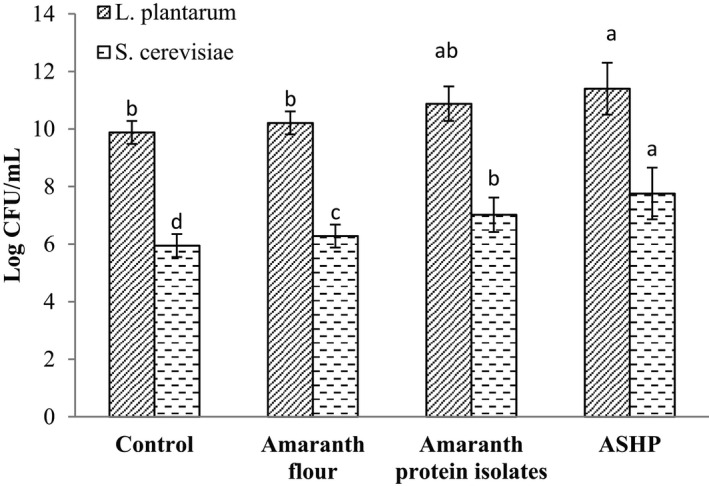
*Lactobacillus plantarum* and *Saccharomyces cerevisiae* growth (log cfu/mL) in vitro with addition of amaranth flour, amaranth protein isolates and amaranth selectively hydrolyzed proteins (ASHP). Results were calculated from mean values of triplicate determinations. Different superscript small letters denote significant differences among treatments (*p* ≤ .05)

The effect of selectively hydrolyzed amaranth proteins on the growth of two key sourdough microorganisms was determined and compared with that of API and AF (Figure 1). The growth of LAB and yeast was influenced by ASHP (*p* ≤ .05). In the other words, ASHP significantly increased the *L. plantarum* growth by 5.3 and 12.2% compared to API and AF, respectively. Partially hydrolyzed shrimp proteins have been shown to increase *L*. *casei* growth (Duan et al., 2011). The study determined the effect of ASHP on the growth of *S*. *cerevisiae* and the highest growth was observed using ASPH (*p* ≤ .05). Control culture medium with API and AF showed lower yeast growth (7.02 and 6.28 log cfu/ml, respectively) compared to the ASHP (*p* ≤ .05). Wheat gluten hydrolysates enhanced yeast growth and ethanol production and increased yeast tolerance to ethanol (Yang et al., 2021). Free amino acids and small peptides have an important role in accelerating the growth of microorganisms and acidification during fermentation. APIs have a complex and folded structure that is inaccessible by. However, a selectively hydrolyzed protein with an unfolded structure can be easily available to microorganisms. Presumably, these peptides are stable and absorbed because of their small size, which is resistant to peptidase (Seppo et al., 2003). The excretion/secretion of yeast proteases into the fermentation environment could hydrolyze larger peptides and proteins into smaller molecules in order to supply the proliferating yeast cells with more available nitrogenous nutrients (Lieske & Konrad, 1994). ASPH with its small size and higher solubility is presumably absorbed more efficiently by LAB and yeast, leading to higher growth compared to protein isolates or AF. Zhang et al. (2011) revealed that small peptides increased LAB growth in comparison with polypeptides and intact proteins, which is consistent with the findings of this study. Based on these data, ASHP could increase the growth of LAB and yeast, more likely influencing sourdough fermentation.

### 3.2 Sourdough properties

**FIGURE 2:**
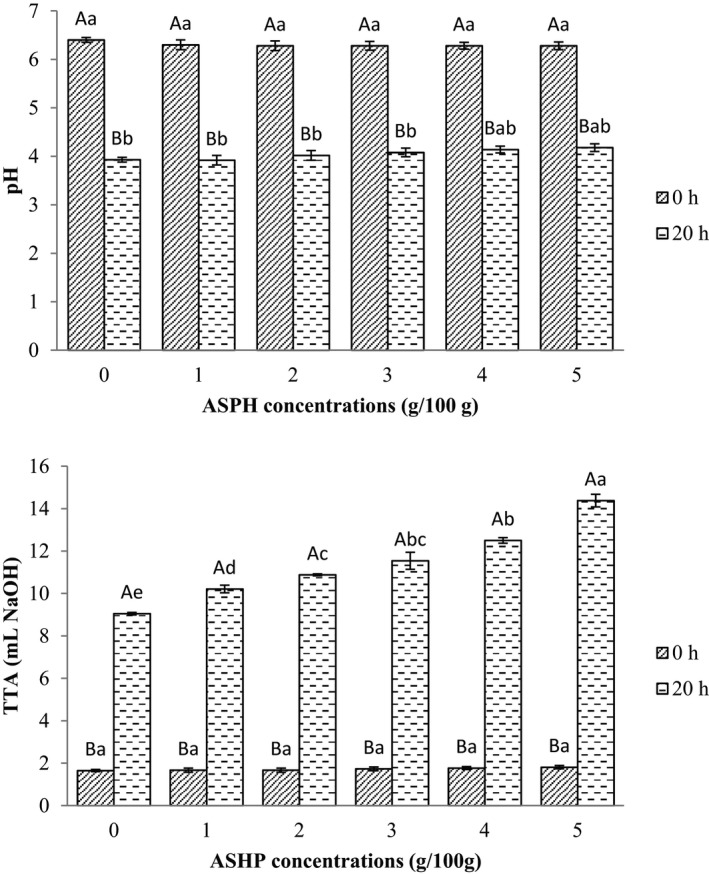
pH and TTA of sourdough after 0 and 20 h of fermentation at 30ºC. ASHP: amaranth selectively hydrolyzed proteins. Results were calculated from mean values of triplicate determinations. Different superscript small letters denote significant differences among treatments (*p* ≤ .05)

The characteristics of the prepared sourdough using ASHP at 0–5 g/100 g were measured (Figure 2) and the initial pH of sourdough ranged from 6.28 to 6.40 (*p* > .05). Sourdough fermented with different concentrations of ASHP after 20 h had pH values from 3.92 to 4.18 and was within the ranges reported in a previous study (Yu et al., 2019). There were no differences in TTA among different sourdoughs at time 0 (*p* > .05). The TTA of all ASPH sourdough was higher than that of control sourdough (*p* ≤ .05). Sourdough with 5% ASHP demonstrated the highest TTA (*p* ≤ .05). The increase of TTA in sourdough is assumed to be due to the higher organic acid produced by LAB during the fermentation process (Jagelaviciute & Cizeikiene, 2021). Duan et al. (2011) reported that shrimp protein hydrolysates increased the growth of *L. plantarum* and lactic acid production in sourdough. Although the higher concentration of ASHP led to the lowest pH while the highest TTA, it increased the acidic taste of the bread. Therefore, bread was produced with a 3% addition of ASHP sourdough. On the other hand, ASPH increased the growth of *L. plantarum* and *S. cerevisiae* in sourdough (Table 1). Sourdough with added ASHP showed higher bacterial and yeast growth compared to common sourdough (*p* < .05). Additionally, higher growth was found by increasing ASHP concentrations in sourdough. Sourdough with 5/100 g ASHP represented the highest growth of *L*. *plantarum* and *S. cerevisiae* at the end of fermentation.

**TABLE 1:** The counts of *Lactobacillus plantarum* and *Saccharomyces cerevisiae* (log cfu/g) in sourdough prepared using amaranth selectively hydrolyzed protein (ASHP) at different concentrations

ASPH (g/100 g)	*S. cerevisiae*	*L. plantarum*
0	4.56 ± 0.05^a^	8.24 ± 0.10^a^
1	4.89 ± 0.08^b^	8.80 ± 0.08^b^
2	5.24 ± 0.13^c^	9.13 ± 0.13^c^
3	5.60 ± 0.13^d^	9.55 ± 0.14^d^
4	5.86 ± 0.07^e^	9.90 ± 0.08^e^
5	6.02 ± 0.08^e^	10.25 ± 0.09^f^

Note

Different small superscript indicate significant difference (*p* < .05).

### 3.3 Characteristics of bread

#### 3.3.1 Physicochemical properties

S‐ASHP sourdough at 2% in bread decreased pH while increasing TTA in comparison with the prepared control bread (Table 2) using common sourdough (CSB, *p* ≤ .05). This was partly attributed to the increased production of organic acids by LAB as fermentation progresses in the dough, leading to decreased pH whereas increased TTA in the bread (Yu et al., 2019). Yu et al. (2019) found that fermentation with specific strains of *L. plantarum* (i.e., NOS7315, MC3, and MC4) led to lower pH values in breads compared to other strains. ASHP significantly increased the growth of *L. plantarum* (Table 1) that has high acidification capacity (Prückler et al., 2015) and amylolytic enzyme activity (Bartkiene et al., 2017), leading to a low pH level in bread. The prepared sourdough prepared with amaranth hydrolysates potentially contributed to higher TTA in the resulting bread. This was coincidental with a higher specific volume (4.57 ml/g) in the fermented bread with S‐ASHP sourdough than that of CSB (3.94 ml/g, *p* ≤ .05). The production of lactic acid and carbon dioxide from glucose by LAB and yeast requires the presence of small chain length peptides and free amino acids (Liu et al., 2018). Therefore, the higher specific volume of the bread made with S‐ASHP sourdough might be due to higher yeast growth and carbon dioxide production (Yu et al., 2019). This bread also demonstrated that a lower staling rate is more likely because of an increase in the loaf specific volume (Arendt et al., 2007). Based on the findings, ASHP increased the growth of *S. cerevisiae* and *L. plantarum* and enhanced sourdough fermentation (Table 1, Figure 2), probably leading to an increase in the specific volume of the prepared bread with S‐ASHP sourdough. S‐ASHP‐B bread had higher moisture content (43%) compared to CSB bread (36.9%, *p* ≤ .05). The effects of sourdough on the properties of dough and bread were assumed to be due to the direct impact of lowering the pH resulting from increased acid production. The activity of flour protease and amylase increases at lower pH, resulting in decreased staling of bread (Arendt et al., 2007). On the other hand, increased proteolytic activities in bread in the presence of peptides cause free water to bind to the gluten network. This indicates that although the moisture content of bread containing peptides is higher, water activity is less and the staling of bread represents a decrease (Arendt et al., 2007). The enthalpy of CSB and S‐ASHP‐B breads at day 1 was 130.26 J/g and 108.79 J/g, respectively. CSB indicated higher enthalpy in comparison with S‐ASHP‐B at day 5 of storage (*p* ≤ .05). A decrease in the staling rate, which was evidenced by bread enthalpy from DSC analysis, has also been reported for breads containing sourdough (Corsetti et al., 1998). Crystallization is the main reason for bread staling (Guo et al., 2020). Particular LAB strains involved in sourdough fermentation contribute to lowering the staling rate of the bread. The produced enzymes by LAB affect the retrogradation properties of the starch, slowing the rate of staling (Fadda et al., 2014). Amaranth peptides have been found to bind with starch, reducing the starch binding with water molecules and lowering starch retrogradation (Karimi et al., 2021). This might explains the lower staling of the S‐ASHP‐B bread.

**TABLE 2:** Effect of different sourdough on pH, TTA, moisture, a_w_, specific volume, and enthalpy of transition (J/g) of breads

Properties	Bread prepared by		Significance
	Modified sourdough (S‐ASHP‐B)	Common sourdough (CSB)	
pH	5.20 ± 0.02	5.29 ± 0.02	*t* = −4.77 sig = 0.009
TTA	4.79 ± 0.06	2.71 ± 0.28	*t* = 12.389 sig = 0.005
Moisture (%)	43.03 ± 1.41	36.99 ± 0.87	*t* = 6.27 sig = 0.003
Aw	0.94 ± 0.01	0.96 ± 0.01	*t* = −3.521 sig = 0.02
Specific volume (mL/g)	4.576 ± 0.06	3.94 ± 0.11	*t* = 8.550 sig = 0.001
Anthalpy1 day ( J/ g)	108.79 ± 0.70	130.26 ± 2.06	*t* = −17.088 sig = 0.00
Anthalpy5 day (J/ g)	163.81 ± 1.90	180.73 ± 1.25	*t* = −12.888 sig = 0.00

Note

*n* = 3. Sig denotes significance. *t* denotes an independent‐samples *t* test.

#### 3.3.2 Textural properties of bread

**FIGURE 3:**
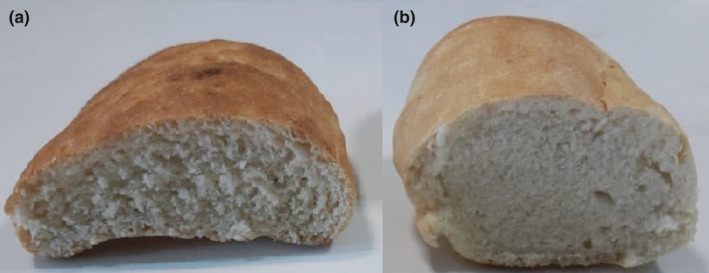
Bread made with S‐ASHP sourdough (a) and common sourdough (b)

S‐ASHP sourdough influenced bread texture compared to CSB (*p* ≤ .05, Table 3). Hardness and chewiness decreased in bread fermented with ASHP‐S sourdough while an increase was found in cohesiveness and springiness compared to CSB (*p* ≤ .05). S‐ASHP sourdough with higher LAB and yeast growth might increase the rate of gas production in bread, leading to a more coherent and flexible bread texture (Figure 3). The textural of bread is highly related to mechanical properties and moisture content (Di Monaco et al., 2015). The competition between hydrolysates with hydrophilic nature and amylose or amylopectin for water molecules led to inadequate starch gelatinization and more heterogeneous crystals as a result of low amylopectin chains mobility and starch retrogradation (Karimi et al., 2021; Niu et al., 2018). The findings suggested that ASHP‐S sourdough has the potential to enhance the texture of the bread.

**TABLE 3:** Textural properties of breads fermented with different sourdough

Properties	Bread prepared by:		Significance
	Modified sourdough (S‐ASHP‐B)	Common sourdough (CSB)	
Hardness(g)	388.33 ± 49.66	544.33 ± 36.69	*t* = −2.597; sig = 0.01
Cohesiveness	0.757 ± 0.03	0.655 ± 0.02	*t* = 17.606;sig = 0.02
Springiness(mm)	0.935 ± 0.01	0.904 ± 0.01	*t* = 12.941;sig = 0.00
Chewiness (g)	282.67 ± 53.51	487.00 ± 46.50	*t* = −7.138; sig = 0.00

Note

*n* = 3. Sig denotes significance. *t* denotes an independent‐samples *t* test.

#### 3.3.3 Sensory properties of bread

Based on the obtained data, S‐ASHP sourdough improved S‐ASHP‐B bread softness, elasticity, and overall acceptance compared to CSB (*p* ≤ .05, Table 4). S‐ASHP‐B bread showed a higher score for flavor and taste in comparison with the prepared CSB suing control sourdough. Small peptides and free amino acids from mussel protein hydrolysates increased bread flavor (Vijaykrishnaraj et al., 2016). Moreover, peptides increase the production of lactic acid and alcohol in sourdough due to higher LAB and yeast growth, resulting in bread with better flavor (Yu et al., 2019). In sourdough, glutamine is converted into glutamate and γ‐aminobutyric acid by the action of *L. plantarum* (Stromeck et al., 2011; Vermeulen et al., 2006) improving bread flavor. The conversion of glutamine to glutamate by the action of *L. reutei* 100‐23 during sourdough fermentation increased bread flavor (Zhao et al., 2015). The higher growth rate of *L. plantarum* and *S*. *cerevisiae* in S‐ASHP sourdough might play a role in the better flavor of the S‐ASHP‐B bread.

**TABLE 4:** Sensory analysis of breads fermented with different sourdough

Properties	Bread prepared by:		Significance
	Modified sourdough (S‐ASHP‐B)	Common sourdough (CSB)	
Color	4.6 ± 0.22	3.8 ± 0.20	*t* = 2.683; sig = 0.01
Elasticity	4.9 ± 0.10	3.2 ± 0.24	*t* = 6.32; sig = 0.00
Taste/flavor	4.8 ± 0.13	3.6 ± 0.16	*t* = 5.692; sig = 0.00
Chewiness	4.6 ± 0.16	3.4 ± 0.22	*t* = 4.366; sig = 0.00
Overall acceptability	4.7 ± 0.15	3.6 ± 0.16	*t* = 4.919; Sig = 0.00

Note

*n* = 3. Sig denotes significance. *t* denotes an independent‐samples *t* test.

#### 3.3.4 Shelf life of bread

The growth of the mold on CSB and S‐ASHP‐B breads stored at room temperature for 9 days was determined in this study. Molds were visible at day 6 of storage in the CSB bread, while no mold growth was observed in S‐ASHP‐B at day 9. Organic acids are produced by the action of LAB during sourdough fermentation, which could inhibit the growth of pathogenic bacteria and fungi and yeast in bread (Jagelaviciute & Cizeikiene, 2021; Kam et al., 2007). *L*. *plantarum* LR 14 produced antimicrobial peptides that inhibited bread spoilage (Gupta & Srivastava, 2014). It was documented that the applied thermophilic LAB for sourdough preparation decreased bread crust surface spoilage and the shelf life of bread was influenced by particular LAB strains (Cizeikiene et al., 2020). Thus, the shelf life of bread made with S‐ASHP sourdough increased compared with the CSB.

## 4 CONCLUSIONS

Overall, ASHP further enhanced sourdough fermentation compared to AF and nonhydrolyzed proteins. In addition, the prepared sourdough using ASHP increased the specific volume and TTA, whereas it decreased the water activity and hardness of S‐ASHP‐B bread compared with the control. Bread with modified sourdough showed a softer texture. Moreover, the sensory properties and shelf life of bread increased with the S‐ASHP addition. The finding indicated the improvement of the quality of bread by sourdough that was prepared with ASHP. Accordingly, ASHP might be useful as the potential natural ingredients of sourdough and wheat bread.

## CONFLICT OF INTEREST

The authors certify that they have no conflict of interests with respect to this manuscript.

## AUTHOR CONTRIBUTION

**Nayyereh Karimi:** Data curation (equal); Formal analysis (equal); Investigation (equal); Writing‐original draft (lead). **Fariba Zeynali:** Conceptualization (equal); Methodology (equal); Project administration (equal); Supervision (equal); Writing‐review & editing (equal). **Mahmoud Rezazad Bari:** Formal analysis (equal); Investigation (equal); Methodology (equal); Writing‐review & editing (equal). **Mehdi Nikoo:** Conceptualization (equal); Investigation (equal); Project administration (equal); Supervision (equal); Writing‐original draft (equal); Writing‐review & editing (equal). **Forogh Mohtarami:** Conceptualization (equal); Data curation (equal); Formal analysis (equal); Writing‐original draft (equal); Writing‐review & editing (equal). **Mehdi Kadivar:** Conceptualization (equal); Methodology (equal); Writing‐review & editing (equal).

## ETHICAL STATEMENT

The care and use of animals and the experimental protocols were approved (No. 952361002–2018‐04‐17) by the animal science committee of Urmia University.

## Data Availability

Data will be made available on responsible request.
